# Differences in the quality of oral anticoagulation therapy with vitamin K antagonists in German GP practices – results of the cluster-randomized PICANT trial (Primary Care Management for Optimized Antithrombotic Treatment)

**DOI:** 10.1186/s12913-019-4372-y

**Published:** 2019-08-01

**Authors:** Cornelia Mertens, Andrea Siebenhofer, Andrea Berghold, Gudrun Pregartner, Lisa-Rebekka Ulrich, Karola Mergenthal, Birgit Kemperdick, Sylvia Schulz-Rothe, Sandra Rauck, Sebastian Harder, Ferdinand Michael Gerlach, Juliana Johanna Petersen

**Affiliations:** 1 Institute of General Practice, Goethe University Frankfurt am Main, Theodor-Stern-Kai 7, 60590 Frankfurt/Main, Germany; 2 Institute of General Practice and Evidence-based Health Services Research, Medical University Graz, Auenbruggerplatz 2/9, A-8036 Graz, Austria; 3 Institute for Medical Informatics, Statistics and Documentation, Medical University Graz, Auenbruggerplatz 2/9, A-8036 Graz, Austria; 4 Institute of Clinical Pharmacology, Goethe University Frankfurt am Main, Theodor-Stern-Kai 7, 60590 Frankfurt/Main, Germany

**Keywords:** Oral anticoagulation, Vitamin K antagonists, General practice, Time in therapeutic range (TTR), Center-specific time in therapeutic range (cTTR)

## Abstract

**Background:**

In Germany, patients receiving oral anticoagulation (OAC) are often treated by general practitioners (GPs), and large proportions of patients receive vitamin K antagonists (VKAs). The quality of OAC in German GP practices, differences between various practices, and improvement potential through implementation of case management, have not yet been investigated satisfactorily.

Based on results of a cluster-randomized controlled trial, we aimed to assess whether OAC quality can be improved, any variations between practices exist and determine practice- and patient-level factors.

**Methods:**

The PICANT trial (2012–2015) was performed in 52 GP practices in Hesse, Germany. Adult patients with long-term indication for OAC received best practice case management in the intervention group. International normalized ratio (INR) values were recorded from anticoagulation passes. The Rosendaal method was used to calculate Time in Therapeutic Range (TTR) at patient level, and mean pooling to obtain center-specific TTR (cTTR) at practice level. The quality of OAC was assessed by TTR and cTTR. Linear model analyses were used to investigate associations between practice−/ patient-level factors and TTR.

**Results:**

Inclusion of 736 patients (49.6% intervention and 50.4% control patients); 690 (93.8%) received phenprocoumon. Within 24 months, the TTR was 75.1% (SD 17.6) in the intervention versus 74.3% (SD 17.8) in the control group (*p* = 0.670). The cTTR averaged 75.1% (SD 6.5, range: 60.4 to 86.7%) in the intervention versus 74.3% (SD 7.2, range: 52.7 to 85.7%) in the control group (*p* = 0.668). At practice level, the TTR was significantly lower in practices with a male physician and certification in quality management. At patient level, the TTR was significantly higher in patients with moderate to high compliance, in men, and in patients that performed self-management. The TTR was significantly lower in patients with certain comorbidities, and who were hospitalized.

**Conclusions:**

The intervention did not effectively improve OAC quality compared to routine care. Quality of INR control was generally good, but considerable variation existed between GP practices. The variability indicates optimization potential in some practices. The demonstrated association between patient-level factors and TTR highlights the importance of considering patient characteristics that may impede achieving high quality therapeutic outcomes.

**Trial registration:**

ISRCTN registry, ISRCTN41847489, registered 27 February 2012.

**Electronic supplementary material:**

The online version of this article (10.1186/s12913-019-4372-y) contains supplementary material, which is available to authorized users.

## Background

Oral anticoagulation (OAC) is indicated for a variety of conditions. Long-term antithrombotic treatment with OAC reduces the risk of thromboembolic events in patients with atrial fibrillation (AF). AF is a common condition, with an estimated 10 million patients suffering from it in Europe in 2014 [1]. Demographic change is expected to lead to an increase in the number of affected persons to 14 to 17 million by 2030 [1]. OAC is also used in patients with mechanical heart valves, thrombosis and pulmonary embolisms [2].

Since becoming available in 2011, prescriptions of direct oral anticoagulants (DOACs) have increased sharply: In 2012, 38 million defined daily doses (DDDs) of DOACs were prescribed in Germany (vs. 389 mio. DDDs of VKAs), while in 2016, 333 mio. DDDs of DOACs were already prescribed (vs. 320 mio. DDDs of VKAs) [3]. DOACs have several advantages, such as more predictable dosing and fewer drug interactions [4]. They are an effective treatment choice for long-term anticoagulation therapy and are now unanimously recommended in cardiology guidelines as a first-line therapy in non-valvular AF. However, some concerns remain [5]. For instance, DOACs have no readily available monitoring marker [6]. Furthermore, DOACs are contraindicated for patients with mechanical heart valves [7] and severe renal dysfunction (defined as creatinine clearance < 15 mL/min) [8].

Vitamin K antagonists have been used and tested in antithrombotic therapy for more than 60 years [9] and lead to much lower treatment costs than DOACs [3]. Previous trials have shown that antithrombotic therapy with VKAs is highly effective in reducing the risk of thromboembolic complications [2, 10]. Nevertheless, a serious risk of adverse thromboembolic and bleeding events is associated with VKAs. This is particularly true when the internationally normalized ratio (INR) values are outside the target range [11]. To achieve beneficial effects while simultaneously minimizing the risk of adverse events, attending physicians should monitor and adjust the dose of patients taking VKAs. Thus, monitoring and the timely adjustment of the treatment regimen is particularly important for these patients. The quality of oral anticoagulation can be determined by the Time in Therapeutic Range (TTR), which is the proportion of time that measured INR values were within the target range. Previous studies have demonstrated that the efficacy of treatment with VKAs is directly related to the TTR, and that an optimal TTR (> 75%) is associated with a lower risk of adverse events [12, 13]. Patient-level factors such as cognitive impairment, poor adherence or individual drug and diet interactions may affect the quality of the therapy [14–16]. In addition, practice-level characteristics may influence the TTR. Center-specific TTR (cTTR) describes the average, annual TTR of patients treated in a medical center, such as a general practice or a specialized clinic. The cTTR can be used to evaluate the quality of oral anticoagulation monitoring in these centers. In recent studies, the cTTR has mainly been calculated for anticoagulation clinics, as these provide care for a large proportion of patients requiring oral anticoagulation in several countries, such as the UK, Italy and Spain. Besides anticoagulation clinics, OAC management is often undertaken by GPs. In Germany, GPs often work independently of each other in small units of mostly one or two physicians and often own the practice in which they work. In this setting, structures and procedures are not comparable to anticoagulation clinics and may be more personalized and limited in organizational and personnel resources. Thus, results of studies in anticoagulation clinics may not be generalizable and valid in these settings. To our knowledge, differences in the quality of treatment with VKAs between individual GP practices in Germany and practice- and patient-level factors associated with the TTR have not yet been studied satisfactorily.

This study is nested in the cluster-randomized controlled PICANT (Primary Care Management for Optimized Antithrombotic Treatment) trial, which was carried out between 2012 and 2015 by the Institute of General Practice, Goethe University Frankfurt am Main, Germany. The trial included 736 patients with a long-term indication for oral anticoagulation in 52 GP practices in Germany. The aim was to investigate whether a best-practice model that includes major elements of case management can improve antithrombotic management in GP practices and reduce thromboembolic and major bleeding events [17]. The trial assessed the quality of VKA therapy at a patient level by determining the TTR. During the monitoring visits at the practices we got the impression that the quality of OAC treatment varies. Therefore, this sub-analysis focused on the quality of OAC on a practice level and investigated associations between TTR and certain practice and patient characteristics. The aims of this study were to:

- assess whether the PICANT intervention was effective in improving the TTR and cTTR
- describe variations in cTTR between practices
- determine practice- and patient-level factors that are associated with the TTR

## Methods

### Study design and population

The PICANT study was an open cluster-randomized controlled trial conducted in 52 German GP practices [18]. The study was approved by the ethics committee (E 191/11) of Frankfurt University Hospital on June 26, 2012. The objective of the PICANT study was to examine whether the application of major elements of case management can strengthen antithrombotic management in GP practices and thus lead to a reduction in thromboembolic and major bleeding events. First, we determined potentially eligible GP practices from a list provided by the Association of Statutory Health Insurance Physicians, with which GP practices must be registered. Afterwards, 568 randomly selected practices received an invitation to participate and study information materials. Eligibility criteria were reviewed for practices interested in participating in the study. Finally, when 52 GP practices were registered, practice recruitment was finished. To fulfil inclusion criteria, practices had to provide health services to persons with statutory health insurance (covering > 90% of the German population) and to have a software system that could identify potentially eligible patients. Patients were included in the study after practice recruitment but before cluster-randomization. For this purpose, each participating practice and members of the study team created a screening list of potentially eligible patients using the practice software system [17]. Using the random number generator function in Microsoft Excel, randomly selected patients from this list were proposed to the GP and the study team, who then decided whether these patients were potential study participants on the basis of the inclusion criteria. When 30 eligible patients had been identified, they received a written invitation from the GP to participate in the study. Once 15 patients had been included, patient recruitment at that practice was stopped. Inclusion criteria for patients were age > 18 years, a long-term indication for oral anticoagulation based on the guidelines valid at the time, and prescriptions for VKAs (coumarins), antiplatelet therapies, or the DOACs Dabigatran or Rivaroxaban (which had already been approved when the study began). Patients were excluded if they had a life expectancy of < 6 months, psychosis, severe sight disorders or auditory defects, alcohol or drug abuse, inadequate German language skills, or if they lived in institutions that did not allow study participation [17].

### Randomization and masking

Randomization took place, once patient recruitment and the baseline assessment had been completed. The web-based randomization tool “Randomizer for Clinical Trials” (www.randomizer.at) was used to randomly assign practices to the intervention or control group in a ratio of 1:1. This was performed by a member of the Institute of General Practice that had no further involvement in the study. Based on the number of inhabitants in the postal area where the practice was located, randomization was stratified using permuted blocks of size 8. For further details, see the published protocol [17].

### Interventions

Prior to randomization, all practices received information materials, including the evidence-based “Anticoagulation” guideline for general practitioners issued by the Guideline Group of the German state of Hesse, and a standardized information pamphlet for patients produced by the German College of General Practitioners and Family Physicians [17]. In brief, the complex intervention included the additional provision of tools and training for healthcare assistants (HCA), information materials and quality circles for general practitioners, and 24-month case management and information materials for patients.

In detail, HCAs took part in an interactive 1-day workshop and were instructed to perform case management and patient training, as well as to evaluate adherence to medication and patient symptoms. They learned to monitor patients regularly using the Coagulation Monitoring List (Co-MoL) [19] and were also encouraged to motivate patients to perform self-management whenever appropriate. Furthermore, GPs were contacted immediately after randomization in order to provide them with further information on case management. As part of the study, three quality circles were conducted with GPs to discuss the practical difficulties of anticoagulation treatment, and individual case reports. The control group received treatment as usual from their GPs, who obtained only the “Anticoagulation guideline” for general practitioners and got no further advice or control visits. For further details on the intervention please see the study protocol [17] and supplementary information of the main study [18].

### Data collection

Data was collected from patients using questionnaires [20–24] and case report forms at three time points - baseline and at follow-ups after 12 and 24 months. An additional file shows an English version of the knowledge test for GPs which was developed for the PICANT study (see Additional file 1). Further data was extracted from the “anticoagulation passes” (*Marcumarpass*). The anticoagulation pass includes patient data, diagnoses, medications, the INR-target range and each individual INR value (with date of measurement), as well as recommended anticoagulation doses. As the pass contains details on individualized treatment plans and dose adjustment, it provides useful information to other treating physicians. Patients’ INR values were obtained from these passes and missing values were added directly from patients’ medical records. Subsequently, all INR values were manually entered into a database and double checked by two different employees at the Institute. From the data collected at baseline, we selected several practice and patient characteristics to investigate possible associations with the TTR. This initially included basic characteristics of GPs and patients, such as age and gender. Furthermore, practice characteristics, such as size and location and professional experience of the GP were included. Additional patient characteristics investigated were amongst others BMI, compliance, several comorbidities and indication for OAC. To also consider the disease course of patients during the study period, we have included hospitalization and the primary endpoint of the PICANT study (defined as combination of all thromboembolic events requiring hospitalization and major bleeding complications) [18] as covariates. The covariates examined are listed in detail in Tables 4 and 5.

### Calculation of TTR and cTTR

Quality of the INR management was considered to be best expressed by the TTR. The TTR was estimated using linear interpolation between the different measurements in accordance with the *Rosendaal method* [25].

We defined “standard” INR target ranges as recommended in current guidelines [18, 26], with a target range of 2.5 to 3.5 in patients with mitral or double heart valve replacement, and 2.0 to 3.0 in other patients. For an additional calculation, we also analysed the “GP-based” target range, which took into consideration the target ranges documented by GPs in case report forms at baseline. For some patients, these GP-based target ranges differed from those generally recommended in current guidelines [26]. Unlike the calculation of the TTR in the main trial [18], INR values that were intentionally outside the therapeutic range – e.g., due to bridging periods – were now excluded from the calculations. As in previous studies (e.g. by Tosetto et al. [27]), the cTTR for each participating practice was calculated as the average TTR of patients at that practice.

### Statistical analyses

TTR and cTTR values were descriptively summarized using mean and standard deviation (SD). Differences between the intervention and control group were assessed by t test for cTTR and by means of a linear mixed model, due to the clustered nature of the data, for TTR. In the latter analysis, practice was considered as a random factor. Practice- and patient-level characteristics are presented either as absolute and relative frequencies or as mean and SD. Linear mixed model analyses were conducted to determine any association between patient and practice characteristics, and the TTR, both for standard and GP-based target ranges. Again, the practice was considered as a random effect in the analyses, and all models were additionally adjusted for the randomization group. Regression coefficients and 95% confidence intervals are presented. The conditional coefficient of determination, R^2^, for generalized mixed models was calculated to assess model fit. A *p* value < 5% was considered significant. SPSS version 25 and R version 3.4.4 were used for the statistical analyses [28, 29].

## Results

### Baseline characteristics

The PICANT study consisted of 736 patients (365 intervention and 371 control patients) from 52 GP practices. Patients were enrolled between July 2, 2012 and Dec 4, 2012. In the intervention group, the mean (standard deviation [SD]) number of participating patients per practice was 14.0 (1.6), while in the control group it was14.3 (1.5). Details on the screening process and characteristics of the sample have been described elsewhere [18, 30].

In brief, practices and patients in the intervention and control groups showed similar characteristics (see Tables 1 and 2, as well as [18]). However, a smaller proportion of intervention practices than control practices had third-party certification in quality management procedures (46.2% vs. 65.4%), and a smaller proportion of intervention practices offered structured courses for patients (42.3% vs. 61.5%). The mean (SD) age of the patients was 74.4 (9.5) years in the intervention vs. 72.8 (9.3) years in the control group. In the intervention group, 52.6% of the patients were male, compared to 53.9% in the control group, and 11.3% of patients performed INR self-management, compared to 13.3% in the control group.

**Table 1:** Baseline characteristics of practices^a^

Characteristics of practices	Intervention (*n* = 26)	Control (*n* = 26)	Total (*n* = 52)
Practice type			
Single-handed practice, no. (%)	11 (42.3)	11 (42.3)	22 (42.3)
Shared or group practice, no. (%)	15 (57.7)	15 (57.7)	30 (57.7)
Third-party certification in quality management for medical practices (e.g. QEP), no (%)^b^	12 (46.2)	17 (65.4)	29 (56.9)
Location of the practice, no. (%)			
Rural (< 20,000 inhabitants)	12 (46.2)	10 (38.5)	22 (42.3)
Provincial (20,000–100,000 inhabitants)	9 (34.6%)	9 (34.6%)	18 (34.6%)
Urban (> 100,000 inhabitants)	5 (19.2%)	7 (26.9%)	12 (23.1%)
Panel size, registered patients per quarter, no. (%)^c^			
500–999	7 (26.9)	1 (3.8)	8 (15.4)
1000–1499	9 (34.6)	11 (42.3)	20 (38.5)
1500–1999	7 (26.9)	5 (19.2)	12 (23.1)
≥ 2000	3 (11.5)	9 (34.6)	12 (23.1)
Main focus of the practice, no. (%)^d^			
Cardiology	13 (50%)	13 (50%)	26 (50%)
Diabetology	13 (50%)	13 (50%)	26 (50%)
Geriatrics	9 (34.6%)	7 (26.9%)	16 (30.8%)
Natural medicine	3 (11.5%)	4 (15.4%)	7 (13.5%)
Structured training courses for patients, no. (%)	11 (42.3)	16 (61.5)	27 (51.9)
Characteristics of GPs			
Male gender, no. (%)	18 (69.2)	16 (61.5)	34 (65.4)
Age, mean (SD)	52.4 (7.7)	49.3 (7.4)	50.9 (7.7)
Knowledge test on OAC for GPs, points, mean (SD)^e^	9.9 (1.6)	9.6 (1.5)	9.7 (1.6)
Years of job experience since medical school, mean (SD)	23.1 (8.1)	20.4 (7.9)	21.8 (8.0)
Participated in a study in the last 5 years, no. (%)^f^	8 (30.8)	11 (42.3)	19 (36.5)
Characteristics of healthcare assistants			
Age, mean (SD)	40.4 (11.8)	37.9 (12.4)	39.2 (12.0)
Years of job experience (including education), mean (SD)	19.3 (10.1)	18.6 (11.7)	19.0 (10.8)

^a^This is a slightly different version of the original table from the main study [24]

^b^The quality management system QEP (Qualität und Entwicklung in Praxen® [Quality and Development in practices]) was developed by the National Association of Statutory Health Insurance Physicians and regional Associations of Statutory Health Insurance Physicians

^c^In Germany, panel size is calculated as the number of patient registrations in a practice over a 3-month period

^d^Practices may have had more than one focus

^e^Self-developed knowledge questionnaire (sum score 0–12) with higher scores indicating greater knowledge about OAC

^f^Including studies conducted by our own Institute and others (e.g., pharmaceutical companies)

**Table 2:** Baseline characteristics of patients^a^

	Intervention (*n* = 365)	Control (*n* = 371)	Total (*n* = 736)
Sociodemographic characteristics			
Age, mean (SD), years^b^	74.4 (9.5)	72.8 (9.3)	73.6 (9.4)
Male gender, no. (%)	205 (56.2)	200 (53.9)	405 (55.0)
Educational attainment, no. (%)			
No educational attainment	54 (15.9%)	38 (11.3%)	92 (13.6%)
Vocational training	2 (0.6%)	0 (0%)	2 (0.3%)
Vocational on-the-job training	145 (42.8%)	153 (45.4%)	298 (44.1%)
On-the-job training combined with school-based education	38 (11.2%)	46 (13.6%)	84 (12.4%)
Education in a technical college	56 (16.5%)	47 (13.9%)	103 (15.2%)
Polytechnic degree	25 (7.4%)	25 (7.4%)	50 (7.4%)
University degree	19 (5.6%)	28 (8.3%)	47 (7%)
BMI, mean (SD)	28.8 (5.1)	29.1 (4.8)	28.9 (5.0)
Smoking, no. (%)			
Non-smoker	185 (51.5%)	205 (56%)	390 (53.8%)
Former smoker	151 (42.1%)	136 (37.2%)	287 (39.6%)
Occasional smoker	9 (2.5%)	11 (3%)	20 (2.8%)
Regular smoker	14 (3.9%)	14 (3.8%)	28 (3.9%)
Migration background, no. (%)	27 (7.4)	24 (6.5)	51 (6.9)
Clinical characteristics			
Long-term indication for oral anticoagulation therapy, no. (%)^c^			
Atrial fibrillation/flutter	302 (82.7)	295 (79.5)	597 (81.1)
Recurrent venous thromboembolism	32 (8.8)	40 (10.8)	72 (9.8)
Recurrent pulmonary embolism	31 (8.5)	30 (8.1)	61 (8.3)
Mechanical heart prosthesis	29 (7.9)	28 (7.5)	57 (7.7)
Intracardiac thrombus	3 (0.8)	4 (1.1)	7 (1.0)
Other indication	33 (9.0)	34 (9.2)	67 (9.1)
Comorbidities, no. (%)^c^			
Ischemic heart disease	133 (36.4%)	106 (28.6%)	239 (32.5%)
Cerebral insult/bleeding	72 (19.7%)	56 (15.1%)	128 (17.4%)
Congestive heart failure	120 (32.9%)	103 (27.8%)	223 (30.3%)
Peripheral arterial occlusive disease (PAOD)	39 (10.7%)	26 (7%)	65 (8.8%)
Arterial hypertension	317 (86.8%)	307 (82.7%)	624 (84.8%)
Renal insufficiency	62 (17%)	63 (17%)	125 (17%)
Diabetes mellitus	119 (32.6%)	135 (36.4%)	254 (34.5%)
Chronic pulmonary diseases	58 (15.9%)	63 (17%)	121 (16.4%)
Diseases of the esophagus, stomach, duodenum	68 (18.6%)	63 (17%)	131 (17.8%)
Malignant tumor	18 (4.9%)	23 (6.2%)	41 (5.6%)
CHA_2_DS_2_-VASc-Score, no. (%)^d^			
> 1	292 (97.0)	282 (95.9)	574 (96.5)
= 1	9 (3.0)	12 (4.1)	21 (3.5)
Antithrombotic medication, no. (%)^e^			
Phenprocoumon	341 (93.4)	349 (94.1)	690 (93.8)
Dabigatran	8 (2.2)	4 (1.1)	12 (1.6)
Rivaroxaban	7 (1.9)	13 (3.5))	20 (2.7)
Aspirin	4 (1.1)	6 (1.6)	10 (1.4)
Other	9 (2.5)	3 (0.8)	12 (1.6)
Last INR within therapeutic target range, no. (%)^f^	240 (69.2)	239 (68.7)	479 (68.9)
INR self-measurement, no. (%)^g^	39 (11.3)	46 (13.3)	85 (12.3)
Patient compliance, no. (%)^h^			
Highly compliant	308 (84.4)	266 (72.1)	574 (78.2)
Moderately compliant	51 (14.0)	86 (23.3)	137 (18.7)
Not compliant	6 (1.6)	17 (4.6)	23 (3.1)

^a^This is a slightly different version of the original table from the main study [24]

^b^Age was calculated from 15/mm/yyyy since the exact birth date was not documented to ensure data privacy

^c^Patients may have had more than one indication, and/or more than one comorbidity

^d^Refers to 595 patients with atrial fibrillation/flutter and available data

^e^Apixaban and Edoxaban had not been approved at the time of the baseline assessment

^f^Only considers patients receiving phenprocoumon; target INR range as defined by GP

^g^Distinction between self-measurement yes and no, dose adjustment not taken into account

^h^Compliance was assessed for each patient by his GP; data available for 369 patients in control group

### TTR and cTTR

**Fig. 1:**
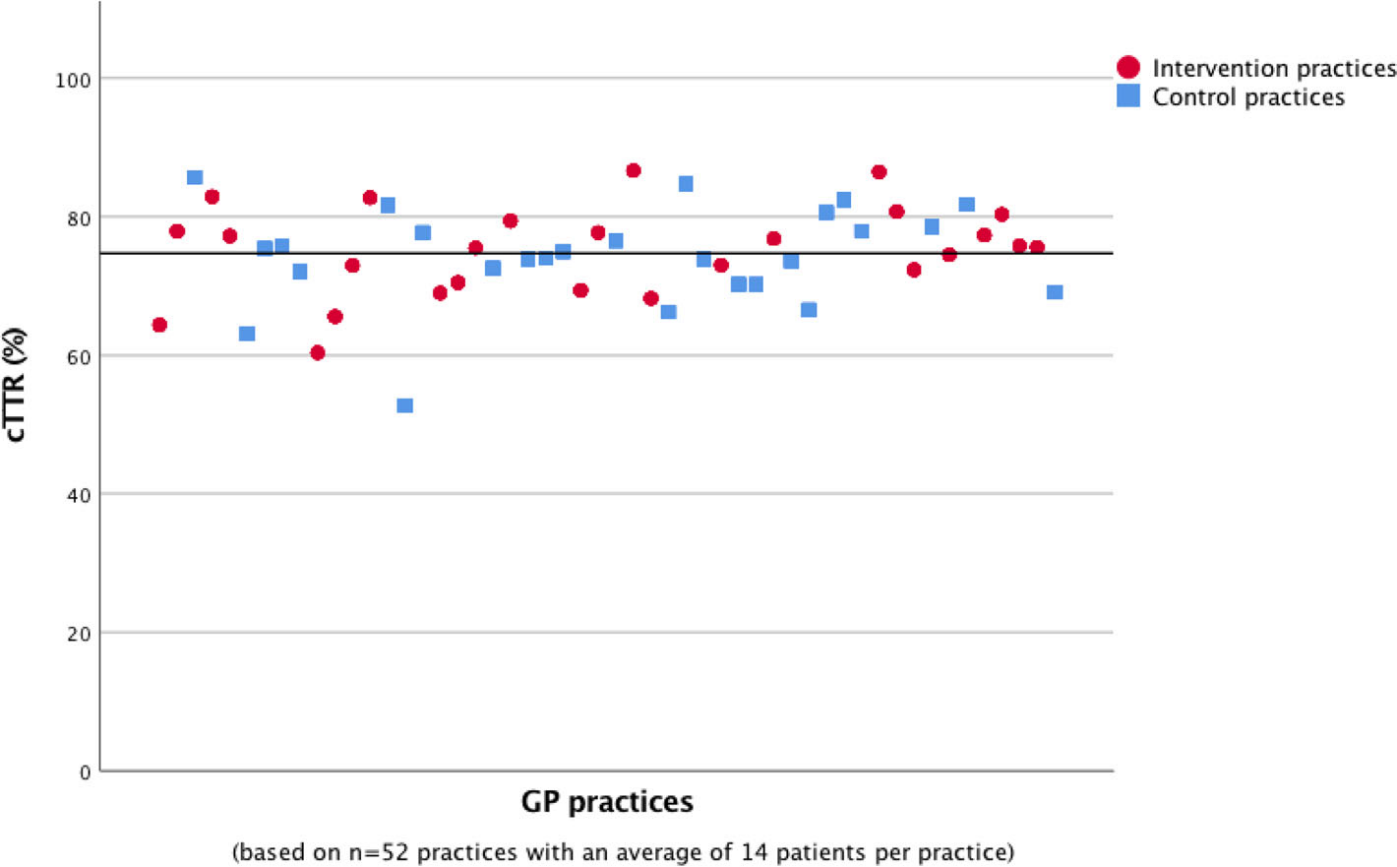
cTTR of the practices based on standard target ranges during the 24-month study period (Calculation of cTTR values excluded bridging periods)

Data on INR measurements were available for 688 patients, 344 from each randomization group. The standard target ranges were 2.0–3.0 for 678 (98.5%) patients and 2.5–3.5 for the remaining 10 (1.5%). GP-based target ranges were more variable with 2.0–3.0 being the most common (657 (95.5%) patients), followed by 2.5–3.5 (11 (1.6%) patients) and 2.0–4.0 (10 (1.5%) patients). For the full list of ranges documented in the anticoagulation passes see Table 3. After 24 months, the TTR based on standard target ranges did not differ statistically significantly between the intervention group (mean TTR 75.1% (SD 17.6)) and the control group (mean TTR 74.3% (SD 17.8)); *p* = 0.670. The mean cTTR was 75.1% (SD 6.5, range 60.4–86.7%) in the intervention group vs. 74.3% (SD 7.2, range 52.7–85.7%) in the control group (*p* = 0.668). Figure 1 shows the variation in the cTTR in the participating practices during the 24-month study period; the cTTR ranged from 52.7 to 86.7%. The average cTTR across both groups is shown as a horizontal line at 74.7%.

**Table 3:** GP-based INR target ranges

Range	Patients (*n* = 688)
1.5–1.8	1
1.8–2.3	1
1.8–2.9	1
1.8–3	1
2–3	657
2–3.5	2
2–4	10
2.5–3.5	11
2.5–4	3
3–4	1

The analyses using “GP-based” INR target ranges showed similar results: Within 24 months, the mean TTR was 75.3% (SD 17.2) in the intervention vs. 74.8% (SD 18.0) in the control group (*p* = 0.787). The mean cTTR was 75.3% (SD 6.4, range 60.4–86.7%) in the intervention group vs. 74.8% (SD 7.6, range 52.7–87.4%) in the control group (*p* = 0.780).

### Associations between practice / patient characteristics and the level of TTR

The results of the linear mixed model analyses based on standard INR target ranges showed that on a practice level, the TTR was significantly lower in practices with a male physician and with certification in quality management. Other factors, such as professional experience of the GP or setting (rural or urban location of the practice) were not statistically significantly associated with the TTR (see Table 4).

**Table 4:** Linear mixed model analyses (TTR calculated according to standard target ranges)^a^ – practice-level covariates

Variables	Regression coefficient	95% confidence interval	*P* value^b^	*R* ^2^
Male gender of GP	−4.07	−7.97; −0.18	**0.041**	0.08
Age of GP, years	0.05	−0.21; 0.32	0.687	0.09
Job experience since medical school, years	0.06	−0.19; 0.31	0.609	0.09
Practice type				
Single-handed practice	Reference			
Shared or group practice	−0.66	−4.56; 3.25	0.737	0.09
Panel size, registered patients per quarter, no. (%)^c^			0.784	0.09
500–999	Reference			
1000–1499	−1.03	−7.12; 5.06		
1500–1999	−3.34	−9.81; 3.14		
> 2000	−2.37	−9.24; 4.51		
Main focus of the practice^d^				
Cardiology	−0.79	−4.65; 3.08	0.684	0.09
Diabetology	−0.43	−4.30; 3.44	0.824	0.09
Geriatrics	−1.13	−5.31; 3.05	0.590	0.09
Natural medicine	−4.02	−9.58; 1.54	0.153	0.09
Third-party certification in quality management for medical practices (e.g. QEP)^e^	−5.12	−8.79; − 1.46	**0.007**	0.09
Knowledge test on OAC for GPs, points^f^	−0.05	−1.30; 1.20	0.933	0.09
Location of the practice				
Rural (< 20,000 inhabitants)	Reference			
Provincial (20,000–100,000 inhabitants)	−3.82	−8.15; 0.52	0.290	0.09
Urban (> 100,000 inhabitants)	−0.58	−5.48; 4.31		

^a^These analyses are based on *n* = 688 patients and the models are adjusted for randomization group

^b^*p* values marked in bold are statistically significant at a significance level of 0.05

^c^In Germany, panel size is calculated as the number of patient registrations in a practice over a 3-month period

^d^Practices may have had more than one focus

^e^The quality management system QEP (Qualität und Entwicklung in Praxen® [Quality and Development in practices]) was developed by the National Association of Statutory Health Insurance Physicians and regional Associations of Statutory Health Insurance Physicians

^f^Self-developed knowledge questionnaire (sum score 0–12) with higher scores indicating greater knowledge about OAC

At a patient level, the models showed that the TTR was significantly higher in patients with moderate to high compliance, in men, and in patients that self-managed their INR values. Here, no distinction was made between self-measurement with and without additional dose adjustment by the patient. A significantly lower TTR was found in patients with a hospital stay during the study period, and in patients with various comorbidities, such as ischemic heart disease, congestive heart failure, chronic kidney disease and chronic pulmonary diseases. A significantly lower TTR was also associated with the occurrence of a primary endpoint of the PICANT study (defined as a combination of all thromboembolic events requiring hospitalization and major bleeding complications documented by GPs in the case report form) during the study period. Factors such as age and educational attainment were not significantly associated with TTR (see Table 5).

**Table 5:** Linear mixed model analyses (TTR calculation based on standard target ranges)^a^ – patient-level covariates

Variables	Regression coefficient	95% confidence interval	*P* value^b^	*R* ^2^
Age, years^c^	− 0.12	− 0.26; 0.02	0.083	0.09
Male gender	2.99	0.38; 5.60	**0.025**	0.10
Educational attainment			0.069	0.11
No educational attainment	Reference			
Vocational training	−47.66	−81.63; −13.68		
Vocational on-the-job training	2.63	−1.53; 6.80		
On-the-job training combined with school-based education	−0.94	−6.12; 4.24		
Education in a technical college	3.19	−1.88; 8.27		
Polytechnic degree	1.78	−4.56; 8.11		
University degree	3.84	−2.58; 10.26		
BMI, units	0.14	−0.13; 0.40	0.318	0.08
Smoking			0.953	0.08
Non-smoker	Reference			
Former smoker	−0.30	−3.04; 2.45		
Occasional smoker	0.96	−7.38; 9.30		
Regular smoker	−2.01	−8.84; 4.82		
CHA2DS2-VASc-Score > 1^d^	3.42	−5.14; 11.98	0.433	0.09
Long-term indication for oral anticoagulation therapy^e^				
Atrial fibrillation/flutter	2.22	−1.06; 5.50	0.185	0.08
Recurrent venous thromboembolism	−0.81	−5.17; 3.55	0.716	0.08
Recurrent pulmonary embolism	−2.12	−6.76; 2.53	0.371	0.08
Mechanical heart prosthesis	−3.27	−8.00; 1.46	0.175	0.08
Intracardiac thrombus	−0.23	−13.22; 12.75	0.972	0.08
Comorbidities^e^				
Ischemic heart disease	−3.42	−6.24; −0.59	**0.018**	0.09
Cerebral insult/bleeding	−2.17	−5.68; 1.35	0.227	0.09
Congestive heart failure	−3.41	−6.31; − 0.51	**0.021**	0.09
Peripheral arterial occlusive disease (PAOD)	−1.83	−6.43; 2.78	0.436	0.08
Arterial hypertension	0.00	−3.64; 3.64	1.000	0.08
Renal insufficiency	−5.76	−9.22; −2.30	**0.001**	0.10
Diabetes mellitus	−1.67	−4.38; 1.05	0.228	0.09
Chronic pulmonary diseases	−5.91	−9.33; −2.49	**0.001**	0.10
Diseases of the esophagus, stomach, duodenum	−0.41	−3.89; 3.07	0.818	0.08
Malignant tumor	−4.31	−9.84; 1.22	0.126	0.09
Compliance^f^				
Not compliant	Reference			
Moderately compliant	6.41	−2.22; 15.04	**0.008**	0.10
Highly compliant	10.87	2.67; 19.06		
Self-measurement (*n* = 648)^g^				
No	Reference			
Yes	5.21	1.74; 8.67	**0.003**	0.10
Hospitalization				
No	Reference			
Yes	−4.51	−7.09; −1.93	**0.001**	0.10
Number of days of hospitalization^h^				
Per day	−0.09	−0.14; −0.04	**0.001**	0.10
Occurrence of primary endpoint^i^	−4.78	−8.85; − 0.71	**0.021**	0.09

^a^These analyses are based on *n* = 688 patients and the models are adjusted via randomization group

^b^p values marked in bold are statistically significant based on a significance level of 0.05

^c^Age was calculated from 15/mm/yyyy since the exact birth date was not documented to ensure data privacy

^d^Reference category “= 1” because no “= 0” exists

^e^Patients may have had more than one indication, and/or more than one comorbidity

^f^Compliance was assessed for each patient by his GP

^g^Distinction between self-measurement yes and no, dose adjustment not taken into account

^h^ “Days in hospital in total” (during the study period)

^i^primary endpoint = combination of all thromboembolic events requiring hospitalization and major bleeding complications, as documented by GPs in the case report form (if more than one event occurred in a patient, the earliest event was considered)

When GP-based INR target ranges were used in the analysis, the results were similar, with only one difference. The gender of the GP was no longer statistically significantly associated with TTR. Detailed results of the linear mixed model analyses using GP-based INR target ranges can be found in Tables 6 and 7. References to results of the main study, that were included in this analysis (Occurrence of primary endpoint and hospitalization) can be seen in Table 8 and in the corresponding paper [18].

**Table 6:** Linear mixed model analyses (TTR calculation based on GP-based target ranges)^a^ – practice-level covariates

Variables	Regression coefficient	95% confidence interval	*P* value^b^	*R* ^2^
Male gender	−3.51	−7.54; 0.52	0.086	0.09
Age of GP, years	0.07	− 0.20; 0.34	0.621	0.09
Job experience since medical school, years	0.08	−0.18; 0.33	0.551	0.09
Practice type				
Single-handed practice	Reference			
Shared or group practice	−1.08	−5.06; 2.90	0.588	0.09
Panel size, registered patients per quarter, no. (%)^c^			0.598	0.10
500–999	Reference			
1009–1499	−2.72	−8.87; 3.43		
1500–1999	−4.83	−11.37; 1.71		
> 2000	− 4.19	−11.13; 2.76		
Main focus of the practice^d^				
Cardiology	−0.41	−4.36; 3.54	0.836	0.09
Diabetology	−0.06	−4.01; 3.89	0.977	0.09
Geriatrics	−0.33	−4.61; 3.95	0.878	0.09
Natural medicine	−4.26	−9.93; 1.40	0.137	0.09
Third-party certification in quality management for medical practices (e.g. QEP)^e^	−5.74	−9.47; − 2.01	**0.003**	0.10
Knowledge test on OAC for GPs, points^f^	0.00	−1.27; 1.28	0.996	0.09
Location of the practice				
Rural (< 20.000 inhabitants)	Reference			
Provincial (20.000–100.000 inhabitants)	−4.14	−8.55; 0.27	0.266	0.10
Urban (> 100.000 inhabitants)	−0.97	−5.96; 4.02		

^a^These analyses are based on *n* = 688 patients and the models are adjusted for randomization group

^b^*p* values marked in bold are statistically significant at a significance level of 0.05

^c^In Germany, panel size is calculated as the number of patient registrations in a practice over a 3-month period

^d^Practices may have had more than one focus

^e^The quality management system QEP (Qualität und Entwicklung in Praxen® [Quality and Development in practices]) was developed by the National Association of Statutory Health Insurance Physicians and regional Associations of Statutory Health Insurance Physicians

^f^Self-developed knowledge questionnaire (sum score 0–12) with higher scores indicating greater knowledge about OAC

**Table 7:** Linear mixed model analyses (TTR calculation based on GP-based target ranges)^a^ – patient-level covariates

Variables	Regression coefficient	95% confidence interval	*P* value^b^	*R* ^2^
Age, years^c^	−0.13	− 0.27; 0.01	0.060	0.10
Male gender	3.14	0.56; 5.72	**0.017**	0.11
Educational attainment			0.161	0.12
No educational attainment	Reference			
Vocational training	−32.54	−66.17; 1.09		
Vocational on-the-job training	3.19	−0.93; 7.31		
On-the-job training combined with school-based education	−0.32	−5.45; 4.80		
Education in a technical college	3.84	−1.19; 8.86		
Polytechnic degree	1.90	−4.37; 8.17		
University degree	5.34	−1.02; 11.69		
BMI, units	0.15	−0.11; 0.42	0.252	0.09
Smoking			0.962	0.09
Non-smoker	Reference			
Former smoker	−0.25	−2.96; 2.47		
Occasional smoker	0.74	−7.51; 8.99		
Regular smoker	− 2.24	−9.00; 4.51		
CHA2DS2-VASc-Score > 1^d^	3.90	−4.61; 12.40	0.368	0.10
Long-term indication for oral anticoagulation therapy^e^				
Atrial fibrillation /flutter	1.90	−1.34; 5.15	0.250	0.09
Recurrent venous thromboembolism	−0.85	−5.17; 3.47	0.699	0.09
Recurrent pulmonary embolism	−2.55	−7.14; 2.04	0.275	0.09
Mechanical heart prosthesis	−2.36	−7.05; 2.32	0.322	0.09
Intracardiac thrombus	−0.93	−13.77; 11.90	0.886	0.09
Comorbidities^e^				
Ischemic heart disease	−3.28	−6.08; −0.49	**0.022**	0.10
Cerebral insult / bleeding	−2.24	−5.72; 1.24	0.206	0.09
Congestive heart failure	−3.77	−6.64; −0.90	**0.010**	0.10
Peripheral arterial occlusive disease (PAOD)	−2.19	−6.75; 2.37	0.346	0.09
Arterial hypertension	−0.25	−3.85; 3.35	0.893	0.09
Renal insufficiency	−5.26	−8.69; −1.84	**0.003**	0.11
Diabetes mellitus	−2.01	−4.70; 0.67	0.141	0.10
Chronic pulmonary diseases	−5.49	−8.88; −2.11	**0.002**	0.11
Diseases of the esophagus, stomach, duodenum	−0.73	−4.17; 2.72	0.679	0.09
Malignant tumor	−3.66	−9.13; 1.81	0.190	0.09
Compliance^f^				
Not compliant	Reference			
Moderately compliant	6.89	−1.64; 15.42	**0.007**	0.12
Highly compliant	11.22	3.11; 19.32		
Self-measurement (*n* = 648)^g^				
No	Reference			
Yes	6.17	2.76; 9.59	**< 0.001**	0.12
Hospitalization				
No	Reference			
Yes	−4.58	−7.13; −2.03	**< 0.001**	0.11
Number of days of hospitalization^h^				
Per day	−0.09	−0.14; −0.03	**0.001**	0.11
Occurrence of primary endpoint^i^	−4.42	−8.44; − 0.39	**0.032**	0.10

^a^These analyses are based on *n* = 688 patients and the models are adjusted via randomization group

^b^*p* values marked in bold are statistically significant based on a significance level of 0.05

^c^Age was calculated from 15/mm/yyyy since the exact birth date was not documented to ensure data privacy

^d^Reference category “= 1” because no “= 0” exists

^e^Patients may have had more than one indication and/or more than one comorbidity

^f^Compliance was assessed for each patient by his GP

^g^Distinction between self-measurement yes and no, dose adjustment not taken into account

^h^“Days in hospital in total” (during the study period)

^i^primary endpoint = combination of all thromboembolic events requiring hospitalization and major bleeding complications, as documented by GPs in the case report form (if more than one event occurred in a patient, the earliest event was considered)

**Table 8:** Considered results of the main study^a^

	Intervention (*n* = 365)	Control (*n* = 371)	Total (*n* = 736)
Occurrence of primary endpoint: patients suffering a thromboembolic or major bleeding event, no. (%)^b^	40 (11.0)	48 (12.9)	88 (12%)
Hospitalized patients, no. (%)	184 (50.4)	209 (56.5)	393 (53.5%)
Days of hospitalization per patient, mean (SD)^c^	12.7 (24.9)	14.5 (24.1)	13.6 (24.5)

^a^This table shows results of the intention-to-treat analysis of the main study, which can be seen in detail in the corresponding paper [24]. It is shown here, because these results were included in the linear mixed model analysis (see Tables 5 and 7)

^b^If more than one event occurred in a patient, the earliest event was counted

^c^Of those patients ever hospitalized

## Discussion

The aim of this analysis was to assess whether the complex PICANT intervention was effective in improving the TTR and cTTR, to describe variations in cTTR between practices, and to determine whether practice- and patient-level factors are associated with the TTR.

According to the results of the main study the PICANT intervention could improve process parameters such as patients’ perceived quality of care and patient and HCA knowledge about OAC [18, 31]. Nevertheless, we found that it did not effectively improve the quality of OAC therapy in terms of the TTR and cTTR. As OAC therapy was generally of good quality in both, the intervention and the control groups, further improvement was perhaps difficult to achieve. In PICANT, the TTR averaged 75.1% in the intervention group and 74.3% in the control group, which is considered good in current guidelines, which recommend a TTR > 70% [26]. In addition, it is higher than the TTR found in previous trials in German GP practices. For example, in a trial by Vormfelde et al., the mean TTR was 66% [32], and in a trial by Mueller et al., the mean TTR was 67.7% [33]. In the thrombEVAL study, which was carried out in GP practices and among ambulatory specialists, the mean TTR was 63.9% [34]. While the TTR in a meta-analysis in the United States was 51% in a primary care setting [35], the Swedish national quality registry for atrial fibrillation and anticoagulation ‘AuriculA’ showed that a high TTR of 80.3% could be achieved in primary care centers [36].

In the PICANT study, the cTTR in the individual GP practices ranged from 52.7 to 86.7%. This rather wide range is in line with results from a previous trial in anticoagulation clinics, in which median cTTR values ranged from 57.7 to 87.7% [37]. We investigated factors at a practice and patient level to determine any association with the TTR. On a practice level, GP practices with third-party certification in quality management (QM) had a lower TTR than practices without such certification. However, a lower proportion of intervention than control practices had third-party certification in quality management (46.2% vs. 65.4%), and the type of certification varied. An examination of the importance of QM certification from the point of view of German GPs showed that the benefits of QM in general practice were viewed critically on account of a tendency towards strict standardization in the treatment of individual patients [38]. Other factors tested at a practice level were not significantly associated with the TTR. In a recent trial in German GP practices, practice characteristics were also unable to explain poor adjustment quality, defined as TTR < 60% [33]. Factors such as differences in the patient collective of a specific practice, or the GP’s response to patient-dependent risk factors, might, for example, lead to poorer quality of therapy.

At a patient level, we found that the TTR was significantly higher in patients with moderate to high compliance, in men, and in patients that self-managed their INR values, while we found a significantly lower TTR in patients with certain comorbidities and with a hospital stay during the study period. Previous studies have described a negative association between female gender and the quality of OAC [39, 40]. A negative association has also been discovered between patients with specific comorbidities and their TTR in previous trials. While ischemic heart disease, congestive heart failure, renal insufficiency and chronic pulmonary diseases were associated with a significantly lower TTR in the PICANT study, a recent trial identified an association with diabetes mellitus and peripheral arterial disease [41]. Schaefer et al. have further shown that the presence of at least two comorbidities, regardless of their nature, is associated with poor quality of OAC, defined as percentage of INR values within target range < 75% [39].

OAC with VKAs is a complex therapy that requires individual dose adjustment and regular INR monitoring. It is therefore plausible that a moderate to high level of compliance in the PICANT study, or adherence as reported in a previous trial [33], was associated with a statistically significantly higher TTR. In PICANT, hospitalization was associated with a significantly lower TTR. However, we were unable to ascertain whether problems in OAC management led to the hospital stays, or whether the TTR was lower as a result of hospitalization, as it may have resulted from a change in the attending physician, or a necessary interruption of OAC due to an invasive procedure. Alternatively, both events may have been triggered by another factor. In PICANT, patients that were self-managing their INR values had a significantly higher TTR. Previous studies have also shown that the INR values of patients that carry out self-management are statistically significantly more likely to be in their therapeutic target range [34, 42, 43].

Compared to standard target ranges, GP-based target ranges were more variable and partly differed from those generally recommended in guidelines. It is necessary to examine critically antithrombotic therapies when the target ranges are outside those specified in guidelines. One reason for a non-standard target range may be that fear of a higher individual risk of bleeding or thromboembolism, encourages GPs to set the target range limits slightly higher or lower than specified in guidelines. Some physicians may also narrow INR target ranges to obtain tighter control of anticoagulation and thus fewer complications. However, a previous study recommended avoiding a narrow INR management strategy since, rather than achieving tighter anticoagulation control, it resulted in a significantly increased incidence of out-of-range INR values and blood draws [44]. Further investigations of practice characteristics associated with high or low TTR values will ​​help in the development of recommendations in primary care. However, the repeatedly demonstrated association between patient-level factors and the TTR underlines the importance of taking into account those patient characteristics that may make it difficult to achieve high quality therapeutic outcomes. Currently, with increased use of DOACs, the importance of VKAs in OAC is changing [3]. However, the discussion on the advantages and disadvantages of VKAs and DOACs is still relevant and the subject of many studies [45, 46]. Choosing the right drug for oral anticoagulation and ensuring the therapy is of high quality continues to be an important challenge for GPs. Despite increased use of DOACs, some patients will still take VKAs because they are indicated in patients with mechanical heart valve replacement or in patients with severe chronic kidney disease. In the future, target group-specific investigations into the quality of therapy may therefore provide further important insights.

### Strengths and limitations

In addition to the large sample size and the intervention period of 24 months, an important strength of the PICANT study is that it depicts the reality of caring for patients with OAC under everyday conditions. For this reason, it is also reassuring that the quality of treatment was generally at a relatively high level already. Another strength is the relatively low loss to follow-up during the study period. After 24 months, 79 of 736 patients (9% in the intervention group vs. 12.4% in the control group) had left the study before it ended either because of death, or the patient’s decision to cancel participation.

There may have been some selection bias, as the proportion of patients self-managing their INR values was higher among participants than among non-participants. One reason for this may be that patients who agree to participate in clinical trials are particularly motivated and therefore more likely to perform INR self-management. However, participants and non-participants showed no relevant differences in terms of age and gender. Hospitalization among participating patients was documented in days, but the INR values measured during such hospital stays could not be ascertained. In 116 (15.8%) patients changed their anticoagulant medication during the course of the study for a variety of reasons. Thus, oral anticoagulation with a given drug could not be monitored over the entire study period in these patients. This fact also reflects the changes in antithrombotic therapy resulting from increased approval of DOACs during the course of the trial. Despite the overall large sample size, the variation in the cTTR between GP practices must be assessed cautiously in light of the limited sample size per practice. Finally, an important limitation in the interpretation of the results are the small values for R^2^ (range 0.08–0.12, see Tables 4, 5, 6 and 7), which suggest that no single factor is able to explain the variance of the TTR. It can be assumed that there is a complex interplay of many individual factors.

## Conclusions

As the quality of OAC was generally high, the intervention resulted in no statistically significant improvement. However, variation between the practices indicates optimization potential in some of them. Nevertheless, a repeatedly demonstrated association between patient-level factors and the TTR underlines the importance of bearing in mind those patient characteristics that may make it difficult to achieve high quality therapeutic outcomes.

## Additional file

Additional file 1: Knowledge test for GPs. The additional file shows an English version of the knowledge test for GPs which was developed for the PICANT study. It was used to evaluate the level of knowledge of participating GPs about oral anticoagulation therapy. (DOCX 220 kb)

## Data Availability

The datasets used and analyzed during the current study are available at Data Archiving and Networked Services (DANS). https://easy.dans.knaw.nl/ui/datasets/id/easy-dataset:112285;jsessionid=9032382CAF7B08281C5C7A7C2A58E465

## Abbreviations

AF: Atrial fibrillation
cTTR: center-specific Time in Therapeutic Range
DDD: Defined daily doses
DOAC: Direct oral anticoagulants
GP: General practitioner
HCA: Health care assistant
INR: International Normalized Ratio
OAC: Oral anticoagulation
PICANT: Primary Care Management for Optimized Antithrombotic Treatment
QM: Quality management
SD: Standard deviation
TTR: Time in Therapeutic Range
VKA: Vitamin K antagonists
