# Supplementary material for: Differences in the quality of oral anticoagulation therapy with vitamin K antagonists in German GP practices – results of the cluster-randomized PICANT trial (Primary Care Management for Optimized Antithrombotic Treatment)
Source: BMC Health Serv Res. 2019 Aug 1;19:539. doi: 10.1186/s12913-019-4372-y (PMC6676561; doi:10.1186/s12913-019-4372-y)
Supplement: Supplementary file 1 — Knowledge test for GPs. The additional file shows an English version of the knowledge test for GPs which was developed for the PICANT study. It was used to evaluate the level of knowledge of participating GPs about oral anticoagulation therapy. (DOCX 220 kb) [file 12913_2019_4372_MOESM1_ESM.docx]

**Knowledge test for GPs Date _ _ _ _ 20 _ _** D D M M Y Y

This test is used to evaluate the level of knowledge of participating GPs about oral anticoagulation therapy.

1. **Which parameters/diseases does the CHA_2_DS_2_-VASc score take into consideration? Multiple answers are possible.**
   1. Arterial hypertension
   2. Renal insufficiency
   3. Age
   4. Diabetes mellitus
   5. Liver disease
   6. Stroke
2. **A 30-year-old woman has a heterozygous factor V Leiden mutation (APC resistance). She smokes 10 cigarettes a day and takes oral contraceptives, which she does not want to stop. Which statements are correct? Multiple answers are possible.**
   1. Oral anticoagulation is indicated.
   2. The woman should be informed by a physician about specific risk situations, such as immobility in case of fractures, infections and long flights.
   3. Her physician should inform her about the risk factors.
   4. An annual blood sample to determine her coagulation profile would be useful.
   5. A family history of the incidence of thromboembolisms should be taken.
3. **Dabigatran (Pradaxa®) is new to the market and may be prescribed in patients with non-valvular atrial fibrillation. Which statements are correct? Multiple answers are possible.**
   1. Taken twice daily, a 150 mg dose reduces the rate of stroke by a good 0.5% per year in absolute terms, compared to Warfarin (a coumarin).
   2. The cost of the therapy is 15-20 times as high as for a therapy with Warfarin (a coumarin).
   3. Dabigatran is contraindicated in case of mild renal insufficiency.
   4. The dose should be reduced when Verapamil is taken simultaneously.
   5. An antidote exists.
4. **What is the usual INR target range in patients with atrial fibrillation that are taking phenprocoumon (e.g. Marcumar®)? Please give only one answer.**
   1. INR: 1.5 – 2
   2. INR: 2 – 2.5
   3. INR: 2 – 3
   4. INR: 2.5 – 3.5
5. **Patients with a mechanical mitral valve are anticoagulated with phenprocoumon (e.g. Marcumar®). What is the correct therapeutic INR target range for such patients? Please give only one answer.**
   1. INR: 2 – 3
   2. INR: 2 – 4
   3. INR: 2.5 – 3
   4. INR: 2.5 – 3.5
   5. INR: 3.5 – 4.5

**Knowledge test for GPs**

**Evaluation**

| **Question number** | **Correct answer(s)** | **Points** |
| --- | --- | --- |
| 1 | a, c, d, f | For each correct answer that was selected, 0.5 points were assigned. 0.5 points were also assigned for each incorrect answer that was not selected. |
| 2 | b, c, e |  |
| 3 | a, b, d |  |
| 4 | c | 2 points were assigned for selecting the correct answer. If an incorrect answer was selected, 0 points were assigned. |
| 5 | d |  |

Sum score: 0-12 points (higher score indicates greater knowledge about oral anticoagulation)
